# Dietary Salt Levels Affect Salt Preference and Learning in Larval *Drosophila*

**DOI:** 10.1371/journal.pone.0020100

**Published:** 2011-06-01

**Authors:** Cheryl Russell, Jan Wessnitzer, Joanna M. Young, J. Douglas Armstrong, Barbara Webb

**Affiliations:** 1 Institute for Perception, Action, and Behaviour, University of Edinburgh, Edinburgh, United Kingdom; 2 Institute for Adaptive and Neural Computation, University of Edinburgh, Edinburgh, United Kingdom; Freie Universitaet Berlin, Germany

## Abstract

*Drosophila* larvae change from exhibiting attraction to aversion as the concentration of salt in a substrate is increased. However, some aversive concentrations appear to act as positive reinforcers, increasing attraction to an odour with which they have been paired. We test whether this surprising dissociation between the unconditioned and conditioned response depends on the larvae's experience of salt concentration in their food. We find that although the point at which a NaCl concentration becomes aversive shifts with different rearing experience, the dissociation remains evident. Testing larvae using a substrate 0.025M above the NaCl concentration on which the larvae were reared consistently results in aversive choice behaviour but appetitive reinforcement effects.

## Introduction

Sodium chloride (NaCl) is important to animals for a variety of physiological functions, including osmoregulation and neural processes. There are a variety of mechanisms that can potentially contribute to maintaining a suitable internal level, including behavioural regulation of salt intake, avoidance of dangerously high salt environments, and metabolic adjustments in the rate of salt excretion [1]. Regarding behavioural regulation, NaCl affects (reflexive) choice or preference behaviours (e.g., *Drosophila* larvae [2], [3], locusts [4], rats [5]). It also acts as a gustatory reinforcer in learning (e.g., crickets [6], [7], locusts [4], [8], *Drosophila* larvae [3]).

Olfactory conditioning with gustatory reinforcement has been established as an important assay in larval *Drosophila* [9] for investigating the neural pathways of learning, e.g. [10]–[13]. NaCl can be positive or negative in its behavioural and reinforcing effects, depending on the concentration [3]. It seems plausible that this may also depend on the current needs or internal state of the animal, but to date there has been relatively little attention paid to the salt content in the rearing medium of larvae used in choice or learning experiments, other than maintaining all animals used within one lab on a constant diet. Yet as noted in [14] “Some of the apparent discrepancies in the literature may arise from differences in the Na+ diet of the different species”. In particular, it might account for a somewhat surprising difference in the unconditioned and conditioned responses observed in [3].

As shown in [3], *Drosophila* larvae respond to low levels of salt with attraction and high levels of salt with aversion. They can also be conditioned to associate odours with salt, showing subsequent attraction to odours paired with low salt and aversion to odours paired with high salt. However, within a specific concentration range, the unconditioned response (UR) exhibited is aversion while the conditioned response (CR) is attraction (compare figure 2 (choice) at the concentration 0.375M NaCl with the same concentration in figure 3B (positive learning) in [3]). In this case, the UR and CR are directly opposite in character; the apparent valence of the US is opposite to its reinforcing effects. While it has long been known in classical conditioning that the CR can differ from the UR [15], it is nevertheless usually expected to be consistent with expectation of the US [16]. But if 0.375M NaCl should be avoided, why should an odour that has been associated with it become more attractive?

Gerber and co-workers also report a similar threshold difference between choice and learning effects for sugars [17]. Niewalda *et al.*. [3] explain the difference as a dissociation in the sensory pathways subserving reflexive behaviour and reinforcement. That is, they suggest the system underlying the reflexive response to salt (UR) is more sensitive than the system underlying reinforcement, so that an (aversively) high concentration to the first system may still appear as an (attractively) low concentration to the second system. They speculate that this may involve different sensory neurons for each pathway, and possibly different receptor gene expression.

The sensory pathways for taste in *Drosophila* larvae are reviewed in [18] and have been investigated in some detail in [19]. Around 90 pairs of gustatory receptor neurons (GRNs) are located across a number of external and internal sensory organs, each organ having several sensilla and multiple receptor types. Although gustatory receptor neurons in *Drosophila* are usually described as four types, responding to sugar (S), water (W), low salt (L1) and high salt (L2) respectively, the actual picture, and the pattern of gene expression, seems rather more complex [20]. Even early reports on the ‘high salt’ receptor neuron note that it seems more activated by acids than salts (e.g. [21]), and Hiroi *et al.*. [22] find manipulation of the ‘bitter’ gustatory receptor (GR) gene *Gr66a* expressed in L2 affects aversive responses to quinine but not to high salt. No GR expression has been shown in L1 (low salt) cells, but Liu *et al.*. [14] found low salt preference in larvae to be abolished after manipulation of *ppk11* expression, and some more complex effects on aversion for *ppk19*; the *ppk* gene family are homologous to the vertebrate epithelial Na+ channel/degenerin family. Low salt levels may also activate the sugar-sensitive neurons [23], and enhance the firing rate of water detecting cells [24].

Although it is known that gustatory receptors project primarily to the suboesophagal ganglion (SOG), tracing of the innervation pathways has largely been based on GR-gal4 lines [19] and hence may not be fully informative about salt taste pathways. From the SOG there are projections to multiple locations including the protocerebrum, the ventral nerve cord, the ring gland (the major endocrine organ of the larvae) and the pharyngeal muscles [25]; and to the mushroom bodies [19] which are thought to be the key location for associative olfactory memory formation [26], [27].

This leaves many possibilities open for different sensory systems (different receptors, different sensory neurons, different sensory organs, or different projection pathways) to be involved in attraction or aversion to salt, and in positive or negative reinforcement by salt. The very simplest possibility, a mechanism that integrates across all the sensory inputs and has a threshold below which salt is good (approach, make positive associations) and above which it is bad (avoid, make negative associations) seems to be ruled out by the results of Niewalda *et al.* [3]. Yet the fact that innate behaviour and associative effects could involve different systems does not itself explain why these systems should have different thresholds: why should an aversive level of salt be rewarding?

One possible explanation is that the sensitivity difference between these pathways is not an innate feature of the larval nervous system but rather reflects a differential response of the two pathways to long-term experience. For example, the salt level preference of the animal in an immediate choice situation may be changed by the salt level of the food it is raised on, while the salt level that has a reinforcing effect may remain genetically fixed; or vice versa. It has been shown that diet containing high levels of salt can affect feeding behaviour [1] in adult *Drosophila*. In locust nymphs, it has been shown that high salt diet affects behaviour (food rejection) [4]. Both studies use rather extreme levels; to our knowledge there is as yet no systematic study in insects of the effects on preference or learning of varying salt levels in food within more reasonable ranges.

Although it seems likely that dietary salt level alters responses to salt, it is not clear in advance what those effects should be. Should a larvae that has a high level of salt in its diet have a decreased threshold for salt preference (as it has no immediate requirement for salt) or an increased threshold (as it has adapted to high salt levels)? Should it find salt less rewarding, or will it need a higher level of salt to experience rewarding effects? The following study aims to answer these questions and thus to test whether independent adaptation of the reflex and reward pathways explains the reported UR-CR dissociation.

## Methods

Larvae were reared on specially prepared food containing different salt concentrations. The food was made by dissolving 10 g of yeast, 10 g of sucrose and 1 g of agarose in 100 ml of distilled water following a minimised NaCl recipe used in [28]. Sodium chloride (NaCl) was added to the required concentration. Larvae were not reared on NaCl concentrations higher than 0.43 M as it has been reported that the emergence rate of many strains falls below 50% in a medium containing more than 0.5 M [28]. This mixture was heated slowly until dissolved and then poured into bottles. The mixture was allowed to solidify and then small holes were put into the food to allow the larvae to burrow easily. Canton-S wild type flies were then put into the bottles and incubated at 25 degrees C on a 12/12 hour light/dark cycle. The larvae were tested at the third instar stage. For comparison, our standard lab food contains, proportionally to 100 ml water, approximately 7 g glucose, 7 g maize, 5 g yeast, 1 g agar, and <1 g antifungal/antibacterial agents (nipagin and proprionic acid).

### Untrained salt preference

The methods used for untrained and trained salt preference follow [3] unless stated otherwise. In each trial, 20–30 larvae were washed in distilled water and placed along the midline of a 90 mm diameter Petri dish with a substrate on one side of pure 2 percent agarose (Pure) and on the other side 2 percent agarose with sodium chloride (NaCl) at various concentrations. The lid was placed on the plate and the larvae were left to move around for 5 minutes, then the number of larvae on each side of the plate was determined (any larvae still within 5 mm of the centre line, or that had burrowed into the agarose or crawled up the sides of the plate are only included in the denominator of equation 1). The preference index was calculated as:
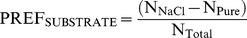
(1)


### Trained odour preference

Larvae were trained by placing them on a Petri dish with either a Pure or NaCl substrate that contained two odour caps (detached lids from microfuge tubes) with either pentyl acetate (denoted PA in the text; Sigma Aldrich, 46022) in 1∶50 dilution with mineral oil or DL-3-octanol (denoted OCT in the text; Sigma Aldrich, 218408) undiluted. Odour balancing was carried out by presenting both odours together, one on each side of the dish, and determining the odour concentrations for which an approximately equal distribution of untrained larvae after three minutes was observed. The lids of the petri dishes were modified with 15 concentrically arranged 1 mm wide holes to improve aeration.

For each experiment, 20–30 larvae were placed on the Pure substrate with one odour. The lid was placed over the dish, and the larvae were allowed to move around on the plate for five minutes. They were then transferred to a plate with the NaCl substrate and the other odour for five minutes. The few larvae that managed to enter the odour cups during this time were unable to exit them, and were discarded from the procedure. The training was repeated three times using fresh plates each time. Half of the experiments paired PA with NaCl (PA+/OCT), the other half paired OCT with NaCl (PA/OCT+). The larvae were then immediately tested for their odour preference by placing them on a plate containing pure agarose with an odour cap containing PA at one side and one containing OCT at the other. After three minutes, the larvae on each side were counted and a preference index calculated as:
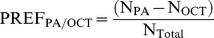
(2)


As for salt preference, any larvae within 5 mm from the centre line were only added to the denominator of equation 2. The performance index was then calculated by combining the results from the reciprocal experiments with those from the alternative odour-substrate pairing:
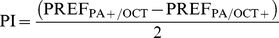
(3)


This provides a measure of how the reinforcer has affected the relative attractiveness of the odours, unbiased by innate preference or learnability of the specific odours used [9].

### Statistics

All graphs and statistical analyses were performed using the free statistical software R [29]. Boxplots show the median (solid line), lower and upper quartiles (Q1 and Q3, box), range (stems) and outliers (circles) defined as data outside the range 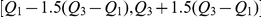. Outliers were not excluded in the following calculations. For all experiments we calculated confidence intervals (C.I.) for the population mean of the preference or performance indices using t-scores from a Student-t distribution with 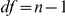. A confidence interval that does not include zero indicates that a mean score is significantly different from zero. For multiple comparisons, the alpha level was adjusted by dividing by the number of comparisons; thus for example, where three scores are compared to zero, the C.I. used is 98.33%. A parametric test is justified as each choice or learning index represents the sum of many binary decisions by individual larvae, hence by the Central Limit Theorem, the distribution should tend towards the normal distribution. Using confidence intervals is appropriate to control for power.

## Results

**Figure 1:**
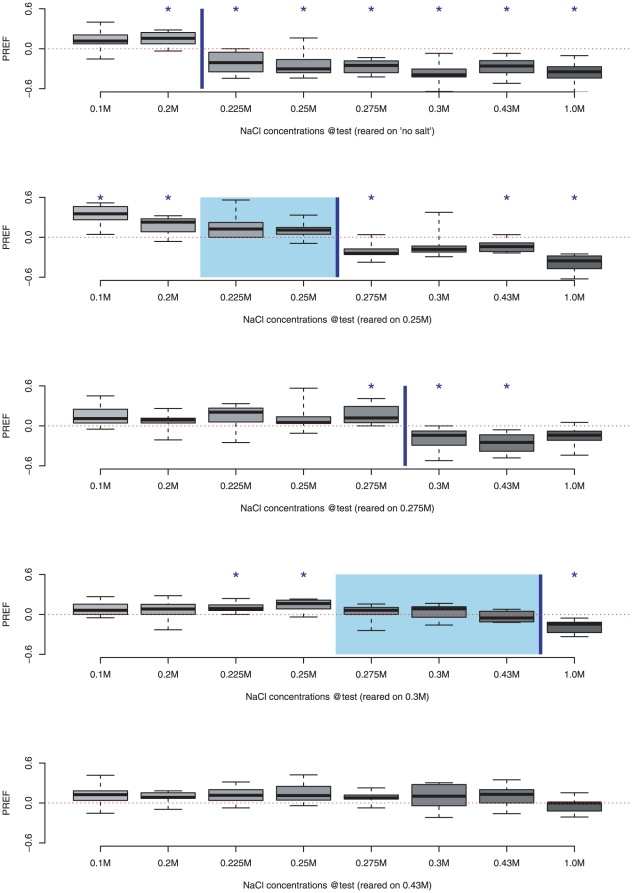
Preference scores for NaCl vs. Pure substrate as the NaCl concentration at test is increased, as a function of NaCl concentration in the food on which the larvae were reared (from top to bottom, food with no added salt, food containing 0.25 M NACl, 0.275 M NaCl, 0.3 M NaCl, or 0.43 M NaCl). Each boxplot represents ten trials, and * denotes a significant difference from zero (the calculated confidence interval of the sample mean with (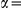
0.05/8) does not include 0). The blue line indicates the lowest NaCl level for which significant aversion occurs, and the start of the shaded area the highest significantly attractive level. The lowest aversive level increases when the NaCl concentration in the food is increased.

We first tested choice behaviours for different rearing conditions as shown in Figure 1. Positive scores indicate attraction to the NaCl substrate, and negative scores indicate aversion. We indicate the concentration value above which the first statistically significant negative score (99.375% C.I. falls entirely below zero) is obtained with a blue line. We find that this crossover point between attraction and aversion increases with the concentration of salt in the food on which the larvae were reared. In general, positive scores are observed up to the level of salt on which the animals were reared, and negative scores are found at higher levels. This suggests the larvae have adapted in some way to the level of salt in their diet. Adaptation is also suggested by the tendency for the attractive response to lower salt levels to become weaker, often not significantly different from zero, as rearing concentration increases, i.e., low concentrations might no longer be detectable. Animals raised on the highest level (0.43 M) show neither clear attraction or aversion, but it is possible this level of salt is compromising to the health of the larvae.

**Figure 2:**
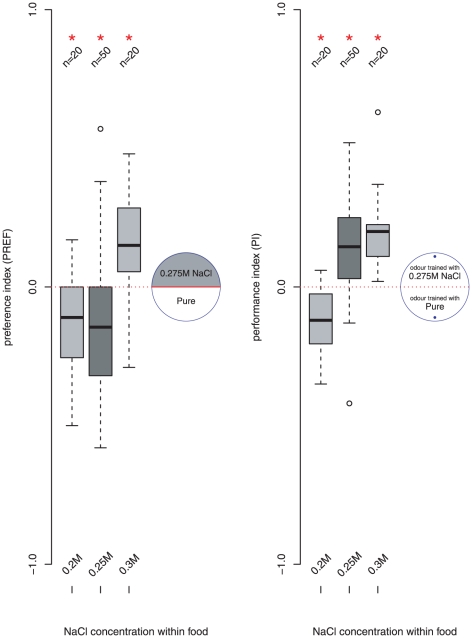
Larvae reared on 0.2 M, 0.25 M or 0.3 M NaCl food tested: (left) for untrained preference between Pure (white) and 0.275 M NaCl (shaded) substrate; (right) for odour preference after associative training with a Pure or 0.275 M NaCl substrate, tested on Pure. * denotes a significant difference from zero (the calculated confidence interval of the sample mean with (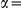
0.05/3) does not include 0, see text). For larvae reared on 0.25 M food (dark grey), the 0.275 M substrate is aversive but the odour associated with it becomes more attractive.

We next compared the choice and learning behaviour of larvae reared with 0.2 M, 0.25 M or 0.3 M NaCl concentration in their food. Each was tested for its initial preference between 0.275 M NaCl and Pure substrates. They were then trained with odours associated to 0.275 M NaCl or Pure, and tested for their odour preference. As can be seen in Figure 2 animals raised on 0.2 M NaCl avoided the 0.275 M NaCl substrate (98.33% CI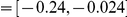, 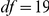) and the preference is shifted away from the odour paired with 0.275 M NaCl substrate (98.33% CI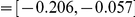, 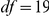). Animals raised on 0.3 M NaCl approach the 0.275 M NaCl substrate (98.33% CI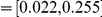, 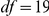) and the preference is shifted towards the odour paired with the 0.275 M NaCl substrate (98.33% CI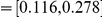, 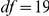). Animals raised at 0.25 M NaCl, however, avoid the 0.275 M NaCl substrate (98.33% CI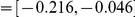, 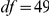) but the preference is shifted towards the odour paired with the 0.275 M NaCl substrate (98.33% CI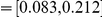, 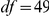), showing the same dissociation between choice and reinforcement properties described in [3]. Note here that the same salt concentration, 0.275 M, has been shown to act both attractively and aversively in the choice paradigm, and to produce both increases and decreases in attractiveness of odours after learning. This reduces the likelihood that the critical concentration at which attraction switches to aversion is a fixed parameter in either system.

**Figure 3:**
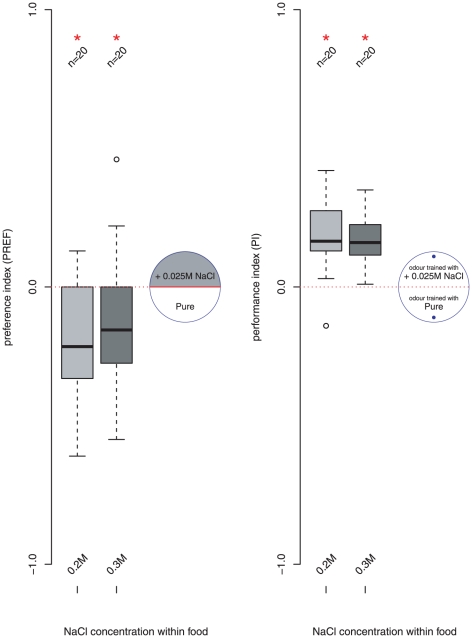
Larvae reared on 0.2 M (light grey) and 0.3 M (dark grey) tested: (left) for untrained preference between Pure (white) and NaCl (shaded) substrate at a concentration 0.025 higher than their food, i.e., 0.225 M and 0.325 M respectively; (right) for odour preference after associative training with the corresponding substrates, tested on Pure. * denotes a significant difference from zero (the calculated confidence interval of the sample mean with (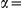
0.05/2) does not include 0, see text). In both cases an aversive substrate has a positive reinforcement effect.

As the aversive choice response appears to be expressed once the salt concentration exceeds the rearing concentration (Figure 1) but an appetitive association is still formed when the level is only 0.025 M higher (Figure 2, larvae raised on 0.25 M NaCl food and tested on 0.275 M) we further tested the larvae raised on 0.2 M or 0.3 M NaCl food concentrations for their choice and learning behaviour using a concentration 0.025 above their food. As shown in Figure 3 larvae raised on 0.2 M NaCl food avoid a 0.225 M NaCl substrate (97.5% CI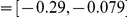, 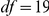) but preferentially approach the odour associated with a 0.225 M NaCl substrate (97.5% CI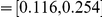, 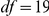). Similarly, larvae raised on 0.3 M NaCl food avoid a 0.325 M NaCl substrate (97.5% CI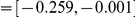, 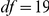) but approach the odour associated with a 0.325 M NaCl substrate (97.5% CI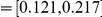, 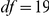). Thus we find that the UR-CR dissociation reported in [3] is replicable for larvae that have different dietary salt experience.

Finally, we note that in Figure 2, the expression of conditioned aversion to the odour paired with 0.275 M NaCl by the animals raised on 0.2 M NaCl food concentration did not show the dependence on the testing substrate reported by [30]. That is, we found a significantly negative performance index when testing on a Pure substrate, whereas [30] only observe expression of conditioned aversion (in their experiments, to odour paired with 0.5 M or 4.0 M NaCl) in the presence of the aversive reinforcer. As they also observe a complementary dependence of the expression of attractive conditioned behaviour (in their experiments, to odour paired with 0.375 M NaCl) on the *absence* of the reinforcer, we repeated our training of the animals raised on 0.25 M NaCl with an odour paired to 0.275 M NaCl, which produces a positive perfomance index when tested on Pure, but this time tested the animals' odour preference on a NaCl substrate. The performance scores are very similar, and there is no significant difference between them (Pure substrate 97.5% CI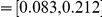, 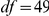); NaCl substrate (97.5% CI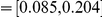, 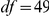).

## Discussion

It appears both preference and reinforcement effects are shifted in a consistent fashion by the feeding experience of the larvae. As a consequence, the CR-UR dissociation observed by Niewalda *et al.*, i.e., the conditioned response of increased approach to an odour that has been paired with a level of salt that produces an unconditioned response of avoidance [3], can be consistently replicated for animals with different feeding experiences; provided a level of salt just above the feeding experience is used for the choice test and odour training. Thus it does not appear that this dissociation is explained by a differential effect of experience on choice and reinforcement.

In particular we note that the same salt level can be either attractive or aversive in a choice test, and can act as either a positive or negative reinforcer, depending on the previous experience of the larvae (figure 2). Consequently it appears that neither the reflex response, nor the reinforcing effects of salt, have a fixed innate threshold. On the other hand there is probably a limited range in which they can be shifted. Niewalda *et al*., in comparing their results to previous studies, suggest the “dose effect curve for choice of salt in larval *Drosophila* is remarkably reproducable” and that the observed change from appetitive to aversive response at around 0.2 M is strikingly consistent with the electrophysiological threshold of the L2 ‘high salt’ receptor neurons at 0.1–04 M. Interestingly our lowest borderline for an aversive response, for larvae reared on a ‘no-salt’ diet, was at a similar value of 0.225 M; but we find larvae reared on increased salt levels required higher levels to show significant avoidance responses.

The fact that an increase in dietary salt leads to an increase in the aversion threshold suggests that the mechanism is one of adaptation rather than intake regulation. That is, larvae do not seek more salty conditions when they lack dietary salt (indeed earlier studies have suggested larvae need only trace levels of salt in their food to survive [28]). Instead, we find that more salt in their diet leads them to tolerate higher salt levels. There are several possibile ways this could occur. There could be a metabolic change to more efficient excretion of salt, with a consequent raising of the required intake to maintain an appropriate level. There could be a change in the salt concentration of the hemolymph and saliva that affects the response of receptors (salivary sodium concentrations have been shown to affect thresholds of taste receptors in rats [31]). There could be active adaptation of the sensors. These possibilities might be expected to have different time courses and thus might be separated by changing high-salt diet larvae onto a low-salt medium for different durations before the choice test.

As the level of salt that is found rewarding seems to remain consistently above the level that is found attractive, as diet is varied, it seems that whatever the mechanism of adaptation, it must also apply, in much the same way, to the sensory system involved in reinforcement. This tends to argue against the complete independence of taste pathways for reflexes and reinforcement. Niewalda *et al.*. [3] consider the possibility that this dissociation is caused by dilution of salt with saliva, if it was assumed that reflexive and reinforcing functions were supported by external and internal taste organs respectively. Thus, an external high-salt sensory neuron could be triggered to produce avoidance behaviour but the diluted salt solution might still activate a low-salt internal sensory neuron. This would not be inconsistent with our results, if we assume both external and internal neurons undergo the same adaptation.

Nevertheless, it remains unclear from the perspective of learning theory why an aversive concentration of salt should have rewarding effects. It is possible to speculate in adaptive terms that a signal indicating where to find a physiologically important substance might be coded by the nervous system as positive for concentrations slightly beyond those considered pleasant under normal circumstances. This suggests a more flexible role for associative conditioning than simple strengthening of a CS-CR response depending on the valence of the US. Important insights into learning in vertebrates have been obtained using ‘devaluation’ experiments [32] in which an attractive US (e.g. food) is associated with a CS, but subsequently the US is made less attractive (e.g. by saturated feeding, or by associating the food with poison). If the animal changes the response to the CS, this is indicative that it has indeed learnt a ‘CS predicts US’ relationship, rather than simply associating the CS with a CR or with the valence of the US. Further experiments would be required to test this in larvae, in particular to test what happens if their preference threshold for salt is altered by changing dietary levels after they have associated it with an odour. However, this may not be straightforward depending on the duration of the memory trace and the duration of dietary experience need to alter preferences.

Although we did not see the same dependency of learned behaviour on the test conditions reported in [30], it is not clear why this difference to their results occurs. Our paradigm follows more closely their training and test procedure than [12], who also failed to observe this dependency. But it was not our intention in this paper to explore this specific effect, which has been very thoroughly and convincingly demonstrated in their work to date (see also Schleyer and Gerber, forthcoming). It remains possible that a factor such as the level of salt in the gut during the test, over which we have no direct control but which may have been altered by our feeding procedures, makes a critical difference.

Our results highlight the critical role that theories of motivation and performance must play in linking mechanisms of associative learning to observed changes in behaviour [33]. Indeed, the fact that a US that produces an aversive UR may nevertheless be a positive reinforcer of an approach CR suggests that the ‘valence’ of a US cannot be unambiguously linked to the reflex response it produces, nor can a CR be unambiguously treated as an indicator of the formation of an association between US and CS. It may be possible to use variants on these simple behavioural experiments to separate some of these issues, and to link them directly to recent results about neural pathways involved in motivational gating of learning behaviour in adult * Drosophila* [34].

## References

[1] Stergiopoulos K, Cabrero P, Davies SA, Dow J (2009). *Salty dog*, an SLC5 symporter, modulates Drosophila response to salt stress.. Physiological Genomics.

[2] Miyakawa Y (1982). Behavioural evidence for the existence of sugar, salt and amino acid taste receptor cells and some of their properties in *Drosophila* larvae.. Journal of Insect Physiology.

[3] Niewalda T, Singhal N, Fiala A, Saumweber T, Wegener S (2008). Salt processing in larval *Drosophila*: choice, feeding, and learning shift from appetitive to aversive in a concentration-dependent way.. Chemical Senses.

[4] Trumper S, Simpson S (1994). Mechanisms regulating salt intake in fifth-instar nymphs of *Locusta migratoria*.. Physiological Entomology.

[5] Contreras R, Kosten T (1983). Prenatal and early postnatal sodium chloride intake modifies the solution preferences of adult rats.. Journal of Nutrition.

[6] Mizunami M, Unoki S, Mori Y, Hirashima D, Hatano A (2009). Roles of octopaminergic and dopaminergic neurons in appetitive and aversive memory recall in an insect.. BMC Biology.

[7] Mizunami M, Matsumoto Y (2010). Roles of aminergic neurons in formation and recall of associative memory in crickets.. Frontiers in Behavioral Neuroscience 4 Article.

[8] Trumper S, Simpson S (1993). Regulation of salt intake by nymphs of *Locusta migratoria*.. Journal of Insect Physiology.

[9] Scherer S, Stocker R, Gerber B (2003). Olfactory learning in individually assayed *Drosophila* larvae.. Learning and Memory.

[10] Selcho M, Pauls D, Han KA, Stocker R, Thum A (2009). The role of dopamine in *Drosophila* larval classical olfactory conditioning.. PLOS One.

[11] Honjo K, Furukubo-Tokunaga K (2005). Induction of cAMP response element-binding protein-dependent medium-term memory by appetitive gustatory reinforcement in *Drosophila* larvae.. Journal of Neuroscience.

[12] Honjo K, Furukubo-Tokunaga K (2009). Distinctive neuronal networks and biochemical pathways for appetitve and aversive memory in *Drosophila* larvae.. Journal of Neuroscience.

[13] Pauls D, Selcho M, Gendre N, Stocker R, Thum A (2010). *Drosophila* larvae establish appetitive olfactory memories via mushroom body neurons of embryonic origin.. Journal of Neuroscience.

[14] Liu L, Leonard A, Motto D, Feller M, Price M (2003). Contribution of *Drosophila* DEG/ENaC genes to salt taste.. Neuron.

[15] Rescorla R (1988). Pavlovian conditioning: It's not what you think it is.. American Psychologist.

[16] Niv Y, Schoenbaum G (2008). Dialogues on prediction errors.. Trends in Cognitive Sciences.

[17] Schipanski A, Yarali A, Niewalda T, Gerber B (2008). Behavioral analyses of sugar processing in choice, feeding, and learning in larval *Drosophila.*. Chemical Senses.

[18] Gerber B, Stocker R, Tanimura T, Thum A (2009). Smelling, tasting, learning: *Drosophila* as a study case.. Results and Problems in Cell Differentiation.

[19] Colomb J, Grillenzoni N, Ramaekers A, Stocker R (2007). Architecture of the primary taste center of *Drosophila melanogaster* larvae.. Journal of Comparative Neurology.

[20] Isono K, Morita H (2010). Molecular and cellular designs of insect taste receptor system.. Frontiers in Cellular Neuroscience 4 Article.

[21] Arora K, Rodrigues V, Joshi S, Shanbhag S, Siddiqi O (1987). A gene affecting the specificity of the chemosensory neurons of *Drosophila*.. Nature.

[22] Hiroi M, Tanimura T, Marion-Poll F (2008). Hedonic taste in *Drosophila* revealed by olfactory receptors expressed in taste neurons.. PLoS One.

[23] Hiroi M, Meunier N, Marion-Poll F, Tanimura T (2004). Two antagonistic gustatory receptor neurons responding to sweet-salty and bitter taste in *Drosophila*.. Journal of Neurobiology.

[24] Chapman R (2003). Contact chemoreception in feeding by phytophagous insects.. Annual Review of Entomology.

[25] Bader R, Colomb J, Pankratz B, Schroeck A, Stocker R (2007). Genetic dissection of neural circuit anatomy underlying feeding behavior in *Drosophila*: distinct classes of hugin-expressing neurons.. Journal of Comparative Neurology.

[26] Heisenberg M (2003). Mushroom body memoir: from maps to models.. Nature.

[27] Gerber B, Tanimoto H, Heisenberg M (2004). An engram found? Evaluating the evidence from fruit flies.. Current Opinion in Neurobiology.

[28] Miyoshi Y (1961). On the resistibility of Drosophila to sodium chloride. I. Strain differences and heritability in D. *melanogaster*.. Genetics.

[29] R Development Core Team (2010). R: A language and environment for statistical computing. R Foundation for Statistical Computing.. ISBN.

[30] Gerber B, Hendel T (2006). Outcome expectations drive learned behaviour in larval *Drosophila*.. Proceedings of the Royal Society B.

[31] Contreras R, Catalanotto F (1980). Sodium deprivation in rats: salt thresholds are related to salivary sodium concentrations.. Behavioral and Neural Biology.

[32] Holland P, Straub J (1979). Differential effects of two ways of devaluing the unconditioned stimulus after Pavlovian appetitive conditioning.. Journal of Experimental Psychology: Animal Behavior Processes.

[33] Berridge K, Robinson T (2003). Parsing reward.. Trends in Neurosciences.

[34] KrashesM, DasGupta S, Vreede A, White B, Armstrong J (2009). A neural circuit mechanism integrating motivational state with memory expression in *Drosophila*.. Cell.

